# Impact on diarrhoeal illness of a community educational intervention to improve drinking water quality in rural communities in Puerto Rico

**DOI:** 10.1186/1471-2458-10-219

**Published:** 2010-04-28

**Authors:** Paul R Hunter, Graciela I Ramírez Toro, Harvey A Minnigh

**Affiliations:** 1 School of Medicine, Health Policy and Practice, University of East Anglia, Norwich UK; 2 CECIA, Inter American University of Puerto Rico, San Juan, Puerto Rico; 3 The Gabriella and Paul Rosenbaum Foundation, Lajas, Puerto Rico

## Abstract

**Background:**

Waterborne disease is a major risk for small water supplies in rural settings. This study was done to assess the impact of an educational intervention designed to improve water quality and estimate the contribution of water to the incidence of diarrhoeal disease in poor rural communities in Puerto Rico a two-part study was undertaken.

**Methods:**

An educational intervention was delivered to communities relying on community water supplies. This intervention consisted of student operators and administrators supervising and assisting community members who voluntarily "operate" these systems. These voluntary operators had no previous training and were principally concerned with seeing that some water was delivered. The quality of that water was not something they either understood or addressed. The impact of this intervention was measured through water sampling for standard bacteriological indicators and a frank pathogen. In addition, face-to-face epidemiological studies designed to determine the base-line occurrence of diarrhoeal disease in the communities were conducted. Some 15 months after the intervention a further epidemiological study was conducted in both the intervention communities and in control communities that had not received any intervention.

**Results:**

Diarrhoeal illness rates over a four week period prior to the intervention were 3.5%. *Salmonella *was isolated from all of 5 distributed samples prior to intervention and from only 2 of 12 samples after the intervention. In the 15 months follow-up study, illness rates were lower in the intervention compared to control communities (2.5% *vs *3.6%%) (RR = 0.70, 95%CI 0.43, 1.15), though this was not statistically significant. However, in the final Poisson regression model living in an intervention system (RR = 0.318; 95%CI 0.137 - 0.739) and owning a dog (RR = 0.597, 95%CI 0.145 - 0.962) was negatively associated with illness. Whilst size of system (RR = 1.006, 95%CI 1.001 - 1.010) and reporting problems with sewage system (RR = 2.973, 95%CI 1.539 - 5.744) were positively associated with illness.

**Conclusions:**

Educational interventions directed both at identified individuals and the community in general in small communities with poor water quality is a way of giving communities the skills and knowledge to manage their own drinking water quality. This may also have important and sustainable health benefits, though further research preferably using a randomised control trial design is needed.

## Background

Waterborne disease outbreaks are still a common occurrence in the United States. In the five years from 2000 through 2004, 63 outbreaks of infectious (or presumed infectious) intestinal disease linked to drinking water were reported [1,2]. Of these outbreaks 25 were associated with independent systems, 22 with community systems and 25 with non community systems. Given that the vast majority of people in the US take their water almost exclusively from community systems, non community and independent systems would appear to pose a substantially greater risk to health for the consumer [2]. One of the weaknesses with the current reporting of waterborne outbreaks in the US is that all community systems are classed together. Within the definition of community are a considerable diversity of systems ranging from the very large (serving >100 000 people) to the very small (≤ 500 persons). There is also diversity in management structures and levels of investment. Within existing published summaries it is often not possible to distinguish between the risk of an outbreak in large or small systems. There is evidence from the United Kingdom that very small systems are much more likely to be contaminated and pose a significantly greater risk to their consumers than do larger systems, reflecting the often poor management and maintenance in place in very small systems [3,4].

Detected outbreaks probably represent only a relatively small proportion of all disease attributable to drinking water, even in developed nations [5]. It is especially likely that small outbreaks in small systems will go undetected [6]. Recent attempts in the US have been made to estimate the burden of disease attributable to drinking water [7]. However, these estimates tend to be primarily influenced by the larger water systems that provide drinking water to the majority of Americans. National estimates of waterborne disease burden do not highlight potential inequalities in illness that may be associated with small (501 - 3.300 persons) and very small (< 500 persons) community and non-community systems.

In the United States and associated territories it is estimated that there are 148,907 small and very small systems serving approximately 39,926,720 consumers. About 42,000 of these systems are publicly-owned and about 20,000 of these are what the United States Environmental Protection Agency (EPA) calls "community systems", i.e., serve the same population year-round and are managed by municipal authorities or by the consumers themselves. In Puerto Rico there are 397 small and very small systems in the inventory of the Health Department, of which only 93 are managed by the Island-wide water and wastewater Authority [8]. There are, however, an unknown number of other water systems that are not recorded. Many of the community systems were built by local residents to provide water to just a few houses and have expanded over the years in a fairly *ad hoc *way as more people have built their homes in these communities. It is not known what burden of disease may be attributable to these systems.

This paper reports a study done during 2005 and 2006 in small and very small drinking water systems in Puerto Rico by the Center for Environmental Education, Conservation and Research of Inter American University of Puerto Rico (CECIA). Some of the deficiencies in management and operation of these systems have been reported earlier by the authors, most notably inconsistent chlorine use [9]. The primary aim of this study was to assess the burden of diarrhoeal disease that could be prevented by improved management and operation of these supplies. In so doing we also intended to develop a minimum estimate of diarrhoeal disease burden attributable to inadequate drinking water quality.

## Methods

### The study area

**Figure 1:**
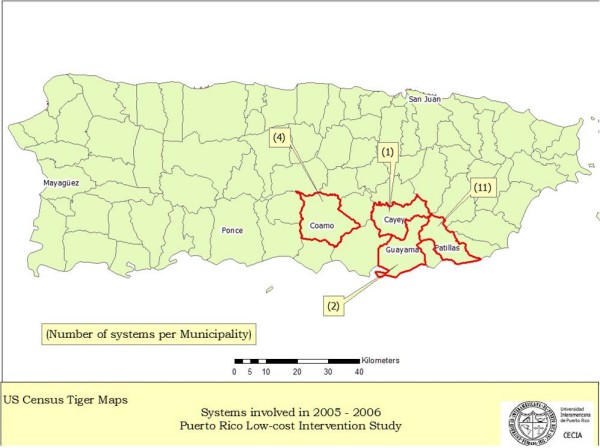
**Location of systems involved in the study**.

**Figure 2:**
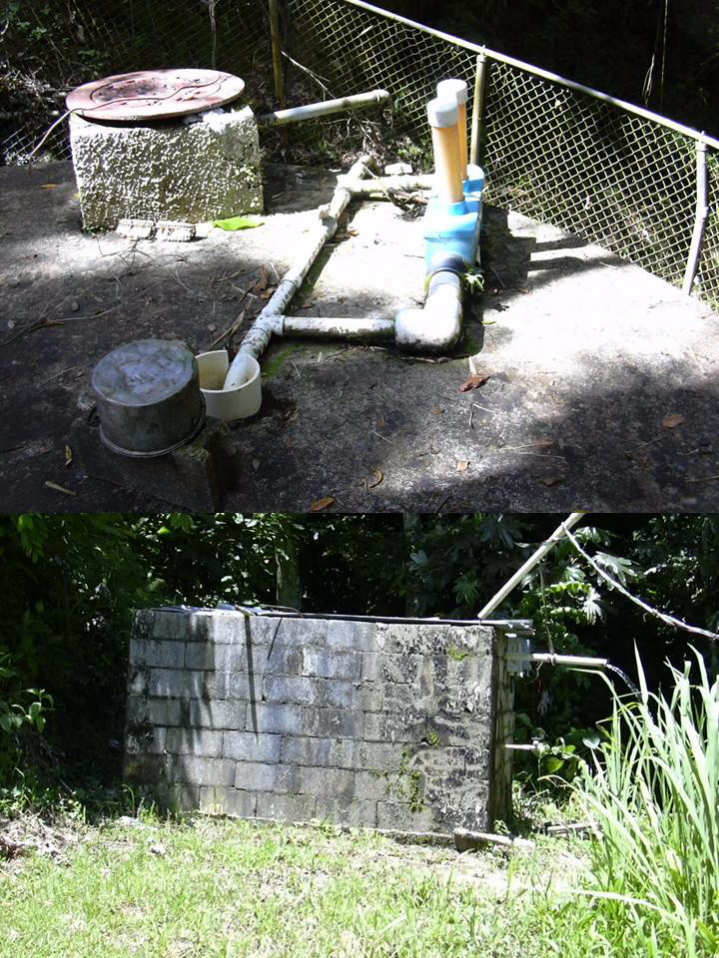
**Examples of water system infrastructure**.

All the systems in the study were small, rural water systems, with ground water (4), surface water (11) or mixed ground and surface water (3) sources. Treatment, if it exists, is limited to chlorination, though almost all people will have a tap connection within the home. The systems are provide water 24 hours a day. The systems are in and serve isolated rural areas with mostly poor residents. The citizens of these economically depressed areas have a median income of $15,000/year (HUD FY2005 Section 8 Income Limits). Whilst this income level is high for many tropical countries, Puerto Rico is politically and economically tied to the United States and the cost of living in Puerto Rico is much the same as elsewhere in the United States. In 2005 the poverty level for families of 4 was $19,971 [10]. Most community members in the productive ages are forced to emigrate in search of work in order to help their families survive. The main economic activity in the area is agriculture. Agriculture is practiced in the same watersheds from which these systems obtain their source water for drinking. Potential sources of contamination are largely unknown to these users and provisions to protect those drinking water sources from microbial and chemical contamination are rarely implemented. The areas in the study are shown in figure 1. Figure 2 shows examples of the nature of the water system infrastructure.

### Intervention

The communities in the intervention study were from the *Cooperativa de Acueductos de Patillas *(CAP), a community-based non-profit organization. Patillas is a municipality with multiple communities some of which have the state managed water supply and others community managed water supplies. In this study we use the term community to refer to people sharing a single water supply that was built and operated by that community. During the intervention phase the cooperative comprised 9 small potable water systems serving approximately 6,000 persons. The CAP is supported by the citizens in the community.

The intervention consisted of meetings with the system management committee (the CAP board) after which two trainee operators and two trainee administrators were identified in each system and enrolled in CECIA professional certification programs. The CECIA programs were designed to provide education and training to persons who were or wanted to be responsible as operators or administrators of small potable water systems. The courses gave a basic understanding of the physics, chemistry and engineering underlying maintenance and safe operation of potable water systems and helped trainees in the programs relate this understanding to the practicalities of the systems they operate or administer. Table 1 lists the modules given as part of the two courses.

**Table 1:** Curricula for Operator and Administrator training courses

Common courses	Hours	Administrator courses	Hours	Operator courses	Hours
Basic commercial Spanish	60	Keyboard and word processing	90	Computer use	90

Basic commercial English	60	Human relations	60	Mathematics for water operators	90

Basic commercial mathematics	60	Information processes in water systems management	120	Biology	90

Environmental health	90	Water systems management	90	Physics	60

Introduction to potable water regulation	90	Document administration	60	Chemistry	60

		Accounting principles	90	Potable water treatment	180

		Internship	215	Operation of water treatment plants	150

				Practical work	405

An essential part of the course was work experience within the trainees' home systems assisting the regular volunteers maintaining the system and administering it. For the operations course this included work towards making disinfection more reliable, cleaning and repairing tanks and water lines, supervising or providing operational assistance, measuring residual chlorine concentrations and suggesting changes in feed rate to system operators. Trainees were supervised on-site by project personnel rather than by existing system volunteers who were not trained operators. An essential part of the theory behind the course was that in their interactions with the existing volunteers so that their new learning would be shared with existing personnel.

A baseline health survey and initial follow-up was done in three of the intervention systems. However, resource constraints meant that this study was limited to the first three systems in which student operators were working, one groundwater and two surface water systems and no control communities were included. The lack of a control group and the non randomised selection of the initial communities would make any interpretation of the findings difficult.

### Epidemiological study

After approximately 15 months a survey was conducted in 8 systems that had received the training intervention and 10 systems that had not. The control systems were selected from the same geographical area as the intervention systems and were chosen to have similar topographical and hydrological characteristics. Each occupied house in the system was visited and invited to participate in the study. The number of participating households is shown in the additional file 1. An introductory questionnaire was administered designed to identify basic demographic data and how many residents had been ill with diarrhoea in the previous four weeks. If anyone within the household reported either diarrhoea and/or vomiting then further questions were administered to determine whether or not they satisfied the case definition. To satisfy the case definition, a case had to report loose or watery bowel movements at least three times in a 24 hour period or any episode of a loose or watery bowel movement with either vomiting or fever. Interviews in each system occurred within a single week and intervention/non-intervention systems were visited roughly alternately.

### Water quality analyses

Samples of raw and distributed water from each system were analyzed for *Salmonella*, total coliform, faecal coliform, *E. coli*, faecal streptococci, heterotrophic plate count (HPC), pH, turbidity, free and total chlorine and temperature. Samples were collected before and after the intervention began. Raw water samples were taken at the source before the distribution network and distributed water samples were taken from a tap in the home that was most distant from the source.

#### Physico-chemical parameters

Bottles for samples were acid-washed and rinsed with tap, RO and RO-DI water three times each. Field measurements of pH and turbidity were made in clean PE bottles rinsed 10 times with the sample. pH was measured both in the field and in the laboratory; field measurements were made with combination electrodes in field packages supplied by VWR^®^. These were calibrated the day of use according to manufacturer instructions. Laboratory measurements were made with a Corning^® ^450 meter and combination electrode calibrated just before use according to manufacturer instructions. All results reported are laboratory pH; field and lab pHs were compared for agreement and no significant differences were found. Air and water temperature were measured in the field using a Fluke^® ^handheld thermometer with a Type-K probe. Turbidity was measured in the laboratory with a nephelometer, calibrated at each use according to manufacturer instructions. In the intervention and initial follow-up study ortho-phosphate, nitrate and nitrite were also analyzed in raw and distributed samples by spectrophotometric methods according to *Standard Methods *[11]. Chlorine measurements were made in the field at the time of sampling using Hach^® ^CN-66 field kits.

### Microbiological methods

#### Standard Indicators and HPC

These were analyzed utilising *Standard Methods *media and incubation procedures. Enumeration to log density was by presence-absence in serial decimal dilutions; from 100 mL for TC, FC and *E. coli *and from 10 mL for fecal streptococci. Dilutions for HPC were from 1 mL and all HPC were spread plates on R2A held in the dark at room T (22-26°C) and counted at 48 and 168 hours. Counts reported are for 168 hours.

#### Salmonella

Approximately 10 L samples were analyzed by an adaptation of *Standard Methods *techniques described elsewhere [12]. This adaptation allowed detection of densities of at least 1 CFU/10 L.

### Data analysis

All statistical analyses were done with SPSS (version 14). For the initial follow-up study the number of person days covered by the questionnaires were calculated for the months January through April. Attack rates per person days were then calculated. For the follow-up study all potential confounding variables were tested to determine whether they differed between the intervention and control communities. All potential categorical variables of interest were tested with either Fisher's exact test or Chi-square for trend. Continuous variables were tested using Student's t test. Further analyses were done using Generalised Estimating Equation with diarrhoea as the dependent variable. All variables significantly different between control and intervention communities at p < 0.2 were entered into the model along with the variable being resident in a intervention community or not. The model was re-run with backward removal of the least significant variable until all variables in the model were p < 0.02.

### Ethical approval

This study was reviewed and approved by the Institutional Review Board of Inter American University of Puerto Rico. All study participants gave informed consent prior to participation.

## Results

### Initial assessment

Water quality samples taken before, during and after the intervention demonstrated the presence of indicators and *Salmonella*. There were no significant associations between any of the standard bacteriological indicators and *Salmonella*, nor between the physico-chemical analytes and either indicators or *Salmonella*. In 8 samples with TC <1/100 mL, i.e., complying with the Total Coliform Rule [13], 7 were *Salmonella *positive, of 11 samples with FC <1/100 mL, 10 were *Salmonella *positive and of 13 samples with *E. coli *<1/100 mL, 11 were *Salmonella *positive.

Finally, there was a significant difference in the occurrence of *Salmonella *before and after the intervention. All of 5 distributed water samples were *Salmonella *positive before the intervention and only 2 of 12 samples afterwards (p = 0.0034 Fishers exact test). All of 5 raw water samples were positive before the intervention as were all of 10 such samples after the intervention.

### Baseline study

The baseline epidemiological study was done in just three of the intervention systems where 89 households were visited between 28^th ^January and 18^th ^February, before the work experience part of the project was started. In these 89 households lived 286 people of whom 75 were <16 years old and 27 < 5 years old. Of these 286 people, 10 (3.5%) reported having had diarrhoea and or vomiting in the previous four week period, equivalent to an annual attack rate of 45.5%.

### Fifteen month follow-up

A total of 922 household questionnaires were submitted which after exclusion of inadequately completed questionnaires (4) and questionnaires from households not on a targeted supply (10) left 908 households. Of these 908 households included in the assessment, 485 households were in the cooperative and received the intervention and 420 were not. Living at these houses were 1291 people in the intervention communities and 1211 people in the non intervention communities. There was no significant difference between the intervention and non-intervention areas for gender or age of the population sampled. There was also no difference between households in the intervention and non-intervention systems for the time the family had lived in the house, the number of bedrooms per house, the number of working cars owned by household members and the education achievement of the main earner in the house, animals owned, drinking water practices or sewage disposal practices. Households in the non-intervention systems had more adults living there (2.33 vs 2.11 p = 0.002), were more crowded (mean number of people per bedroom 1.05 vs 0.95; p = 0.007). Households in the intervention group were also more likely to be part of a smaller water system (mean number of occupied houses attached to system 66 vs 172 in the non-intervention group, p = 1.7 × 10^-90^), to own fewer rabbits and dogs but more birds. (additional file 1, Tables 2 and 3)

**Table 2:** Comparison of possible confounding scalar variables between control and intervention systems.

	Intervention			Control			P
	**N**	**Mean**	**Std Dev**	**N**	**Mean**	**Std Dev**	

Children in house	485	0.55	0.89	423	0.65	0.967	0.095

Adults in house	485	2.11	1.04	423	2.33	1.14	0.002

How long lived at address	481	29.4	21.9	415	30.7	22.9	0.407

How many bedrooms	485	2.98	0.97	423	2.93	0.9	0.443

How many working cars	485	1.42	1.06	423	1.47	1.18	0.449

Crowding	484	0.95	0.57	420	1.05	0.54	0.007

Size of system	485	171.61	91.74	423	65.98	20.22	1.7 × 10^-90^

Age	1288	37.9	22.8	1242	37.1	22.9	0.43

**Table 3:** Comparison of possible confounding nominal variables between control and intervention systems,

		Intervention	Control	P
Gender	M	647	592	0.118

	F	639	662	

Education of main bread winner	Elementary or less	96	95	0.192

	Secondary	254	195	

	High education	134	130	

Problems with sewage	N	441	397	0.056

	Y	41	22	

Problems with neighbours sewage	N	446	385	0.755

	Y	34	27	

Dogs	N	185	194	0.014

	Y	300	226	

Cats	N	341	312	0.204

	Y	144	109	

Rabbits	N	41	18	0.01

	Y	443	404	

Cattle	N	30	24	0.739

	Y	454	399	

Sheep	N	6	6	0.811

	Y	479	417	

Goats	N	10	8	0.854

	Y	475	415	

Pigs	N	24	24	0.626

	Y	461	399	

Birds	N	187	134	0.029

	Y	297	289	

Horses	N	54	48	0.909

	Y	431	374	

The overall diarrhoeal rate within four weeks before the visit in the intervention area was 2.5% compared to 3.6% in the non-intervention systems. Using a generalised estimating equation (GEE) to control for possible clustering within system and within families this was not statistically significant, the corrected rate ratio being 0.58 (95%CI 0.29 to 1.19). Children in intervention and non-intervention systems experienced greater absolute disease risk compared to older age groups and children in the non-intervention system also experienced a higher relative risk compared to children in the intervention system though in neither case was this statistically significant (table 4).

**Table 4:** Diarrhoeal incidence rates in intervention and non-intervention systems with Rate Ratio from Generalized Estimating Equation Poisson regression accounting for possible clustering within systems and within households.

Age group	Intervention systems		Non-intervention systems		Relative risk	Lower 95% CI	Upper 95% CI
	**N**	**Attack rate (%)**	**N**	**Attack rate (%)**			

All ages	1291	2.5	1211	3.6	0.588	0.291	1.186

< 5 years	61	3.3	70	8.6	0.382	0.077	1.895

5 to 15 years	202	3.0	200	4.5	0.657	0.228	1.891

> 15 years	1025	2.3	972	2.9	0.687	0.317	1.483

In the final GEE model living in an intervention system was associated with a marked reduction in relative risk of illness (RR = 0.318; 95%confidence intervals 0.137 - 0.739) (Table 5). Also in the final model were size of system (RR = 1.006, 95%CI 1.001 - 1.010) and reporting problems with sewage system (RR = 2.973, 95%CI 1.539 - 5.744). Owning a dog was negatively associated with illness (RR = 0.597, 95%CI 0.145 - 0.962).

**Table 5:** Final model showing relative risk of diarrhoeal disease associated with intervention and key possible confounders

Predictor variable		RR	Lower 95% CI	Upper 95%CI	P
Intervention system	N	1			

	Y	0.318	0.137	0.739	0.008

Number of occupied houses attached to system		1.006	1.001	1.010	0.014

Reported problems with sewage system	N				
	
	Y	2.973	1.539	5.744	0.001

Owns dogs	N				

	Y	0.597	0.145	0.962	0.034

Some 70 water quality samples were taken during or before the visits. These samples were collected from taps at participating households in the system, usually a tap in one of the houses most distant from the source. The results of key parameters are shown in table 6. There were no significant differences in any of the microbiological or chemical parameters between intervention and non-intervention systems.

**Table 6:** comparison in water quality indicators between intervention and non-intervention systems

Parameter	Non-intervention (n = 41)		Intervention (n = 28)		P
	**Mean**	**Standard deviation**	**Mean**	**Standard deviation**	

Turbidity	1.80	2.16	5.18	12.15	0.157^a^

pH	7.44	0.41	7.45	0.25	0.834^a^

Temp/°C	26.63	1.99	27.13	1.71	0.287^a^

Total chlorine	0.21	0.42	0.47	0.72	0.061^a^

Free chlorine	0.15	0.36	0.61^c^	0.98	0.026^a^

					

	**Absent**	**Present**	**Absent**	**Present**	

Salmonella/L	8	13	6	8	0.778^b^

Total coliforms/100 ml	15	25	14	12	0.191^b^

*E. coli*/100 ml	27	13	18	8	0.883^b^

Faecal streptococci/100 ml	27	14	15	11	0.501^b ^'

^a ^T test

^b ^Chi squared

^c ^Mean free chorine higher than mean total chlorine due to missing total chlorine results.

## Discussion

Care must be taken in the interpretation of our findings in this study. In particular, the number of systems included in the study was not large and the choice of intervention and non-intervention systems was not random. On the other hand, as far as could be assessed, potential confounding variables were accounted for in the final model. Nevertheless, it is certainly possible that the difference in illness rates identified in this study could be due to some other unknown factor.

If it is the case that the difference in illness rates in the intervention and non-intervention communities are due to the intervention alone then our findings would suggest that drinking water is the major cause of diarrhoeal illness in the poor communities of Puerto Rico served by community potable water systems. However, even after the intervention, water quality in the intervention areas still does not achieve full compliance with current US standards, in particular treatment requirements under the Surface Water Treatment Rule [14], as amended, and the Total Coliform Rule [13]. Furthermore, because the only water treatment was chlorination, disease due to more chlorine resistant pathogens such as *Cryptosporidium *would not be affected and may still add to the disease burden in intervention communities.

The intervention described in this study would still not bring these systems up to generally accepted minimum drinking water standards. Drinking water in the intervention systems will still not comply with current regulations. These communities are unable to afford the cost of full compliance. Waiting to implement improvements that would make these systems fully compliant would take years and allow a substantial ongoing and preventable disease burden with significant economic costs on the poorest communities. In this project, effective technical assistance and capacity development achieved the most important step toward compliance - understanding the purpose of responsible water system management and operation. Our work suggests that it is not necessary to wait for interventions that ensure full compliance with current drinking water standards to have a positive impact on public health. In other words it is more important to do something than to wait for the perfect solution.

Much recent research on preventing waterborne disease in poor rural communities has focussed on designing and implementing various in-home or point-of-use treatment devices [15]. Whilst many of these interventions have been shown to be effective in the short term, there are serious concerns about their longer term sustainability [16]. These concerns are mainly around the completeness of community coverage and their long-term continued use. Indeed, the evidence is that for some of these interventions there may be no public health gain after the first few months [17]. It is likely that continuing, relatively costly public education campaigns would be required to maintain compliance and continued public health benefits. Because our approach required the identification and training of a relatively few individuals within the community such continuing education costs will be much smaller.

This project was conceived and implemented before the general promulgation by the World Health Organization of the water safety plan approach (WSP) [18]. The water safety plan approach moves the water safety paradigm from end product testing to encouraging a better understanding and management of the points in the water treatment and delivery process that where failures could increase the risk to public health. To-date there have been few examples of its application to very small systems [19], though the WSP is an approach that would fit very well with the type of educational intervention we have described here.

A further point was that approximately 2/3 of the people included in the study were adults. There has been some suggestion that adults who have lived in contact with contaminated water for many years do not suffer increased risk as a result of this exposure, because of acquired immunity [20,21]. This study suggests that at least in these types of water system ill health effects from contaminated water persist throughout life.

In the intervention program system administrators were asked to nominate members of their community for the program and 17 operator and 14 administrator students were enrolled. Of the 17 students in the operator training program 14 graduated and all of these took the operator certification examination - the first time operators from community systems did this in Puerto Rico. Of these, 7 passed the examination at the highest level these included the first woman operator certified in Puerto Rico (and 3 failed by a single point). In this intervention we also noted some unintended impacts. Most particularly the community people that were trained to look after their systems developed self confidence and transferable skills. Most of these students have subsequently obtained wastewater treatment operators licenses on their own initiative. All the operator students were unemployed at the start of the program and all are now employed in water and/or wastewater systems. Of the administrator students 12 graduated; all but one were unemployed at the start of the project and all now work in either potable water or related industries. All these students have been actively recruited by both corporate water treatment operators or the Island-wide Water and Wastewater Authority. In addition the existing volunteer operators generally welcomed the assistance and the extra hands for routine chores.

## Conclusions

In conclusion, our work shows that interventions aimed at supporting poor communities in taking responsibility for their own systems can have significant and sustained public health benefits. Even when the intervention will not achieve water fully compliant with current standards, there can be major beneficial impacts on public health. Our work would also indicate that water and sanitation problems in poor communities in Puerto Rico -- and probably in similar communities elsewhere in the United States --may still be the major driver of diarrhoeal disease. Drinking water safety in people reliant on very small systems represent a major issue for the public health of people reliant on them for their drinking water, the impact of which may have more serious potential outcomes than just acute diarrhoeal disease [22]. Given that that many people reliant on such supplies may not have the knowledge or wealth to improve their own water supplies, even in relatively wealthy countries, contaminated rural water supplies represents a major issue for environmental justice and drinking water [23].

## Competing interests

PRH is Chair of Science advisory council to Suez Environment, Paris, Chair of Board of Directors of Institute of Public Health and Water Research, and has done consultancy for Danone Beverages. All other authors have no competing interests to declare.

## Authors' contributions

All authors contributed to the study design, GR and HM managed the data collection, PRH undertook analyses and wrote the first draft of the paper. All authors read and approved the final manuscript.

## Pre-publication history

The pre-publication history for this paper can be accessed here:

http://www.biomedcentral.com/1471-2458/10/219/prepub

## Supplementary Material

Additional file 1 **Supplemental table**. System households in follow-up study and brief description of system source and distribution network. Click here for file

## References

[B1] Craun MF Craun GF Calderon RF Beach MJ Waterborne outbreaks reported in the United States J Water Hlth 2006 4 Suppl 2 19 30 10.2166/wh.2006.016 16895084

[B2] Liang JL Dziuban EJ Craun GF Hill V Moore MR Gelting RJ Calderon RL Beach MJ Roy SL Surveillance for waterborne disease and outbreaks associated with drinking water and water not intended for Drinking - United States, 2003-2004 MMWR 2006 355 SS12 31 58 17183231

[B3] Smith A Reacher M Smerdon W Adak GK Nichols G Chalmers RM Outbreaks of waterborne infectious intestinal disease in England and Wales, 1992-2003 Epidemiol Infect 2006 134 1141 9 10.1017/S0950268806006406 16690002 PMC2870523

[B4] Yip Richardson H Nichols G Lane C Lake IR Hunter PR Microbiological Surveillance of Private Water Supplies in England - The impact of environmental and climate factors on water quality Water Res 2009 43 2159 68 10.1016/j.watres.2009.02.035 19303126

[B5] Naumova EN Christodouleas J Hunter PR Syed Q Temporal and spatial variability in cryptosporidiosis recorded by the surveillance system in North West England in 1990 - 1999 J Water Hlth 2005 3 185 196 16075943

[B6] Nichols G Hunter PR, Waite M, Ronchi E Using existing surveillance data Drinking Water and Infectious Disease: Establishing the Links 2002 CRC Press, Boca Raton 131 41

[B7] Messner M Shaw S Regli S Rotert K Blank V Soller J An approach for developing a national estimate of waterborne disease due to drinking water and a national estimate model application J Water Hlth 2006 4 201 40 10.2166/wh.2006.024 16895092

[B8] USEPA Lee K SDWISFED PWS Inventory, FY2005 2006 Washington, DC, US EPA http://www.epa.gov/safewater/data/getdata.html

[B9] Minnigh HA Ramírez Toro GI Regulation and Financing of Potable Water Systems in Puerto Rico, A Study in Failure in Governance 2004 Good Water Governance for People and Nature: What Roles or Law, Institutions, Science and Finance, Dundee, Scotland, UK, American Water Resources Association and International Water Law Research Institute, University of Dundee

[B10] U.S. Bureau of the Census, Current Population Survey, Annual Social and Economic Supplements at http://www.census.gov/hhes/www/poverty/histpov/histpovtb.html

[B11] American Public Health Association Standard Methods for the Examination of Water and Wastewater 1998 20 Washington, DC, APHA, AWWA & WEF

[B12] Herson DS Nichols C Verville K Stromberg T Bright A Ramírez Toro GI Martinez L Ramirez Z Minnigh H Occurrence of Salmonella spp. in Small Potable Water Systems in the Tropics Correlated with Microbiological Indicators of Water Quality - Presentation Number Q-324. 104th General Meeting, Atlanta, GA, American Society for Microbiology 2005

[B13] Total Coliform Rule (TCR), 54 FR 27544-2 June 29, 1989. See EPA 816-F-01-035 2001 54 No 124 http://www.epa.gov/safewater

[B14] The Surface Water Treatment Rule (40CFR141.70-141.75), The Interim Enhanced Surface Water Treatment Rule(40CFR141.170-141.175), The Filter Backwash Rule (40CFR141.76) and The Long Term 1 Enhanced Surface Water Treatment Rule (40CFR141.500-141.571) http://www.epa.gov/safewater/mdbp/pdfs/qrg_mdbp_surfacewatertreatment_convent_direct.pdf

[B15] Fewtrell L Kaufmann RB Kay D Enanoria W Haller L Colford JM Water, sanitation, and hygiene interventions to reduce diarrhoea in less developed countries: a systematic review and meta-analysis Lancet Infect Dis 2005 5 42 52 10.1016/S1473-3099(04)01253-8 15620560

[B16] Arnold B Arana B Mäusezahl D Hubbard A Colford JM Jr Evaluation of a pre-existing, 3-year household water treatment and handwashing intervention in rural Guatemala Int J Epidemiol 2009 38 1651 1661 10.1093/ije/dyp241 19574492 PMC2786251

[B17] Hunter PR House-hold water treatment in developing countries comparing different intervention types using meta-regression Env Sci Technol 2009 43 8991 8997 10.1021/es9028217 19943678

[B18] Davison A Howard G Stevens M Callan P Fewtrell L Deere D Bartram J Water Safety Plans: Managing drinking-water quality from catchment to consumer 2005 World Health Organization, Geneva

[B19] Mahmud SG Shamsuddin SkAJ Feroze AM Davison A Deere D Howard G Development and implementation of water safety plans for small water supplies in Bangladesh: benefits and lessons learned J Water Hlth 2007 5 585 597 10.2166/wh.2007.045 17878569

[B20] Frost FJ Roberts M Kunde TR Craun G Tollestrup K Harter L Muller T How clean must our drinking water be: The importance of protective immunity J Infect Dis 2005 191 809 814 10.1086/427561 15688300

[B21] Swift L Hunter PR What do negative associations between potential risk factors and illness in analytical epidemiological studies of infectious disease really mean? Eur J Epidemiol 2004 19 219 23 10.1023/B:EJEP.0000020453.84296.f6 15117114

[B22] Hunter PR Pond K Jagals P Cameron J An assessment of the costs and benefits of interventions aimed at improving rural community water supplies in developed countries Sci Total Environm 2009 407 3681 3685 10.1016/j.scitotenv.2009.03.013 19344935

[B23] Pontius FW Environmental Justice and Drinking Water Regulations JAWWA 2000 92 3 14 16,18,20,104

