# Supplementary material for: Impact on diarrhoeal illness of a community educational intervention to improve drinking water quality in rural communities in Puerto Rico
Source: BMC Public Health. 2010 Apr 28;10:219. doi: 10.1186/1471-2458-10-219 (PMC2876105; doi:10.1186/1471-2458-10-219)
Supplement: Additional file 1 — Supplemental table. System households in follow-up study and brief description of system source and distribution network. [file 1471-2458-10-219-S1.DOC]

Table. System households in follow-up study and brief description of system source and distribution network

| System | Inter­vention group | Total house­holds in system | Total occu­pied house­holds in system | Partici­pated in study | Person refused to partici­pate | No one was at home | Vacant  House­hold | % of occu­pied house­holds included in survey | Notes on source and distribution system |
| --- | --- | --- | --- | --- | --- | --- | --- | --- | --- |
| 1 | Y | 29 | 29 | 26 | 3 |  |  | 90 | Surface water distributed in a mix of PVC and cast iron pipes, mostly 2” and smaller with some 4”. |
| 2 | Y | 26 | 24 | 23 |  | 1 | 2 | 96 | Well water distributed in ductile iron, most 4”; most less than 25 years old. |
| 3 | N | 90 | 72 | 48 | 4 | 20 | 18 | 67 | Surface water; mostly 2” PVC for distribution. |
| 4 | N | 95 | 83 | 21 | 6 | 56 | 12 | 25 | Surface water; mostly 2” and smaller PVC. |
| 5 | Y | 316 | 290 | 140 | 32 | 118 | 26 | 48 | Surface (95%) and well (5%). PVC, galvanized and cast/ductile iron, mostly 4” and 2”. |
| 6 | N | 86 | 80 | 45 |  | 35 | 6 | 56 | Groundwater; mostly PVC. |
| 7 | N | 16 | 14 | 9 | 5 |  | 2 | 64 | Surface and groundwater water; galvanized and PVC, all 2” and smaller. |
| 8 | N | 37 | 29 | 19 | 3 | 7 | 8 | 66 | Surface and groundwater (surface is “emergency” but often in use). Very long (>4 km) 4” PVC line from source. |
| 9 | N | 40 | 33 | 28 |  | 5 | 7 | 85 | Surface, galvanized and PVC distribution, most 2” and smaller. |
| 10 | Y | 14 | 14 | 9 | 1 | 4 |  | 64 | Groundwater; 4” and 2” ductile and PVC distribution; most newer than 20 years. |
| 11 | N | 47 | 44 | 43 |  | 1 | 3 | 98 | Surface water. Galvanized and PVC with most 2” and smaller. |
| 12 | Y | 48 | 48 | 45 | 2 | 1 |  | 94 | Surface water; PVC and galvanized most 2”, some 4”. |
| 13 | N | 85 | 82 | 69 | 6 | 7 | 3 | 84 | Groundwater with 2” and 4” PVC and ductile iron, most newer than 25 years. |
| 14 | N | 68 | 62 | 41 | 4 | 17 | 6 | 66 | Groundwater with suspected surface water influence. 4” and 2” PVC and ductile distribution. |
| 15 | Y | 141 | 135 | 112 | 17 | 6 | 6 | 83 | Technically both surface and groundwater; though well never in service. 4” and 2 smaller PVC and ductile iron, some galvanized. Much 4” newer than 5 years. |
| 16 | Y | 208 | 184 | 130 | 16 | 38 | 24 | 71 | Technically both surface and groundwater; well no longer in service. Ductile iron and PVC, most 4”. |
| 17 | N | 83 | 83 | 79 |  | 4 |  | 95 | Groundwater with PVC and ductile, most 2” and 4”. |
| 18 | N | 45 | 41 | 21 | 1 | 19 | 4 | 51 | Surface and groundwater.  PVC, galvanized and some cast/ductile, most 2” with 4” iron. |
| Total |  | 1474 | 1347 | 908 | 100 | 339 | 127 | 67 |  |
